# Supplementary material for: Entrustable Professional Activity 10: Case Simulation and Assessment—STEMI With Cardiac Arrest
Source: MedEdPORTAL. 2016 Dec 23;12:10517. doi: 10.15766/mep_2374-8265.10517 (PMC6440413; doi:10.15766/mep_2374-8265.10517)
Supplement: Supplementary file 1 — A. Simulation Case.docx B. Visual Stimuli.docx C. Case Assessment Rubric.docx D. STEMI Management Presentation.pptx [file mep-12-10517-s001.zip › D. STEMI Management Presentation.pptx]

## Slide 1
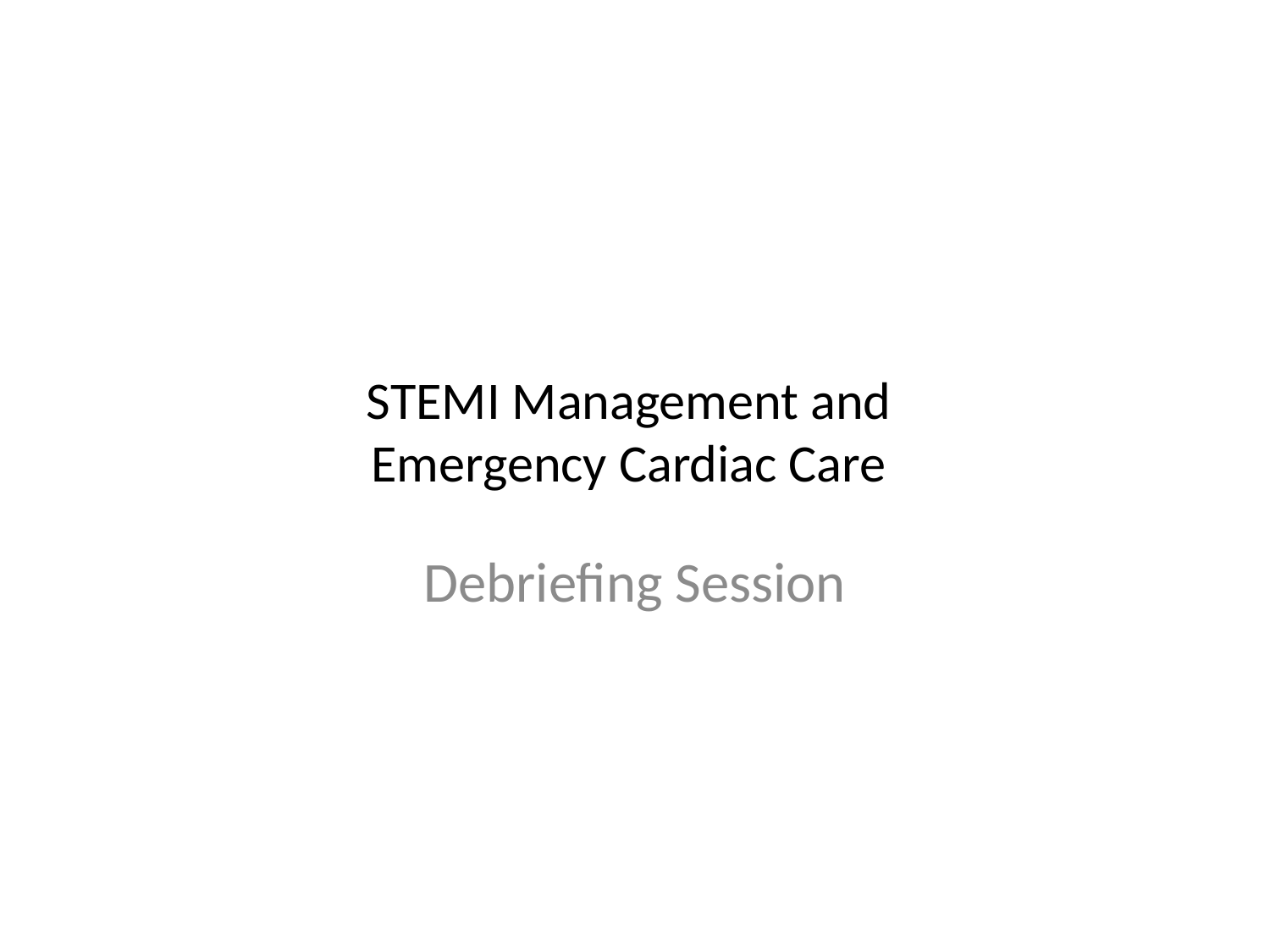

# STEMI Management and Emergency Cardiac Care
Debriefing Session

## Slide 2
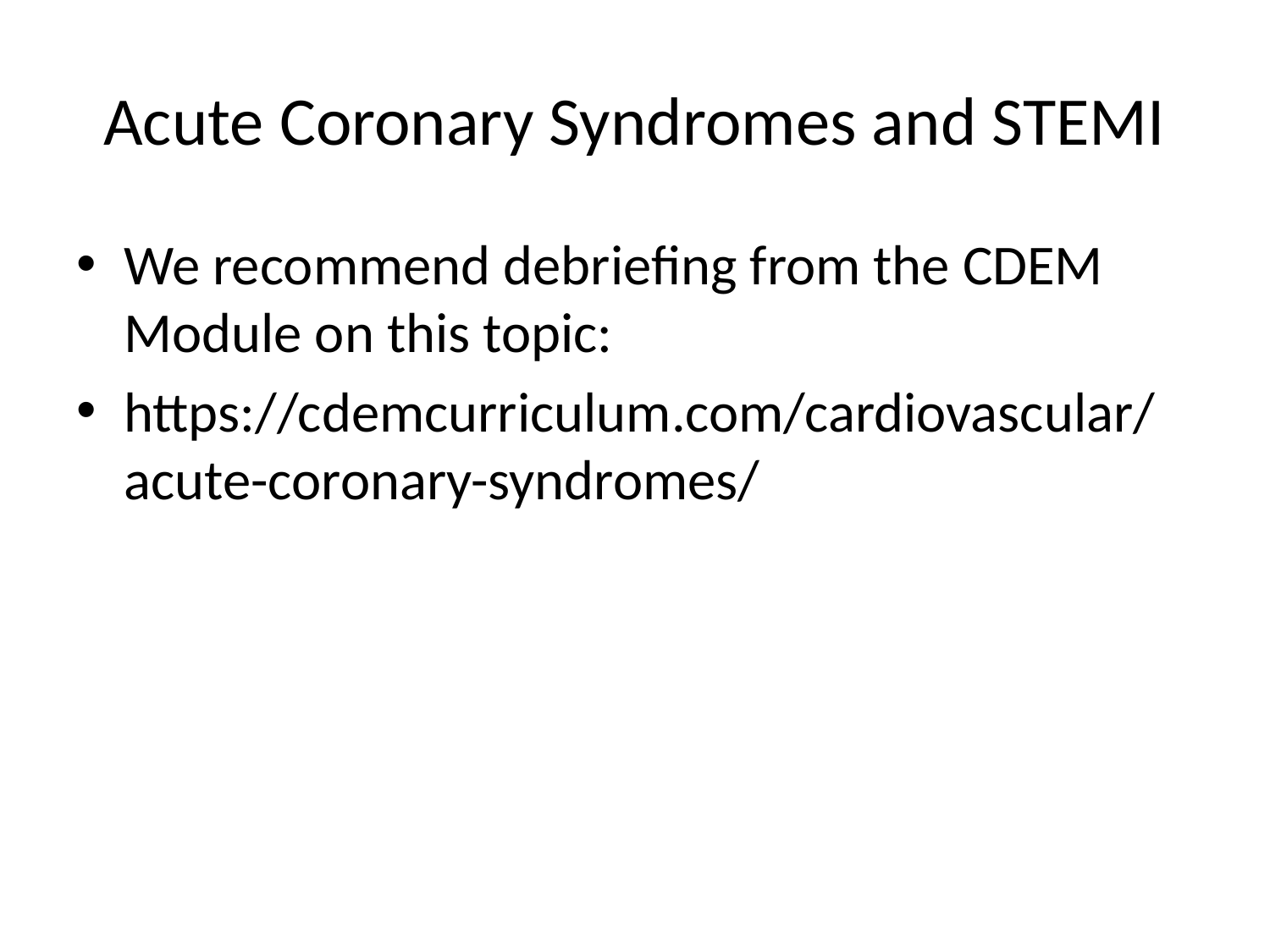

# Acute Coronary Syndromes and STEMI
We recommend debriefing from the CDEM Module on this topic:
https://cdemcurriculum.com/cardiovascular/acute-coronary-syndromes/

## Slide 3
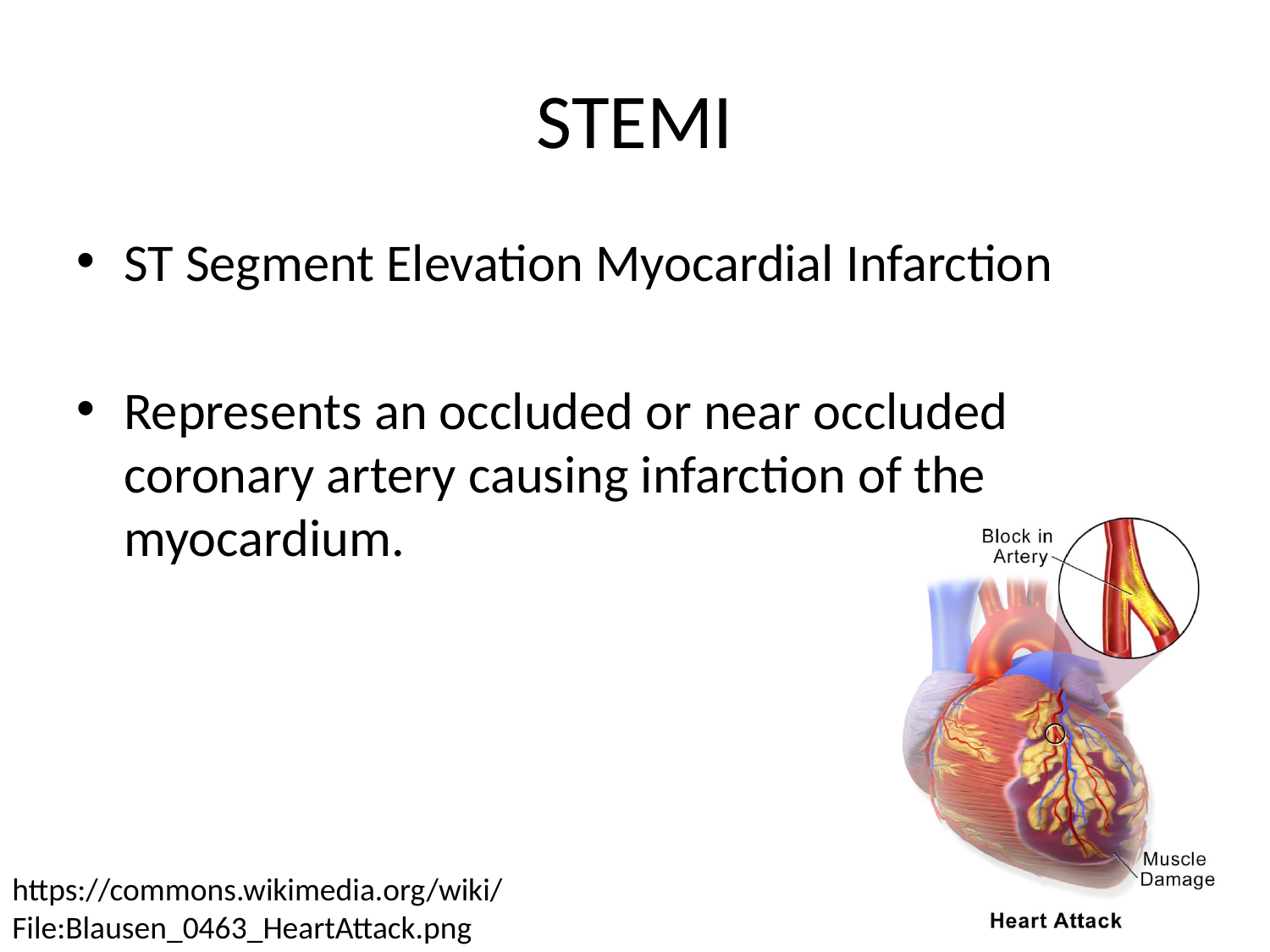

# STEMI
ST Segment Elevation Myocardial Infarction
Represents an occluded or near occluded coronary artery causing infarction of the myocardium.
https://commons.wikimedia.org/wiki/File:Blausen_0463_HeartAttack.png

## Slide 4
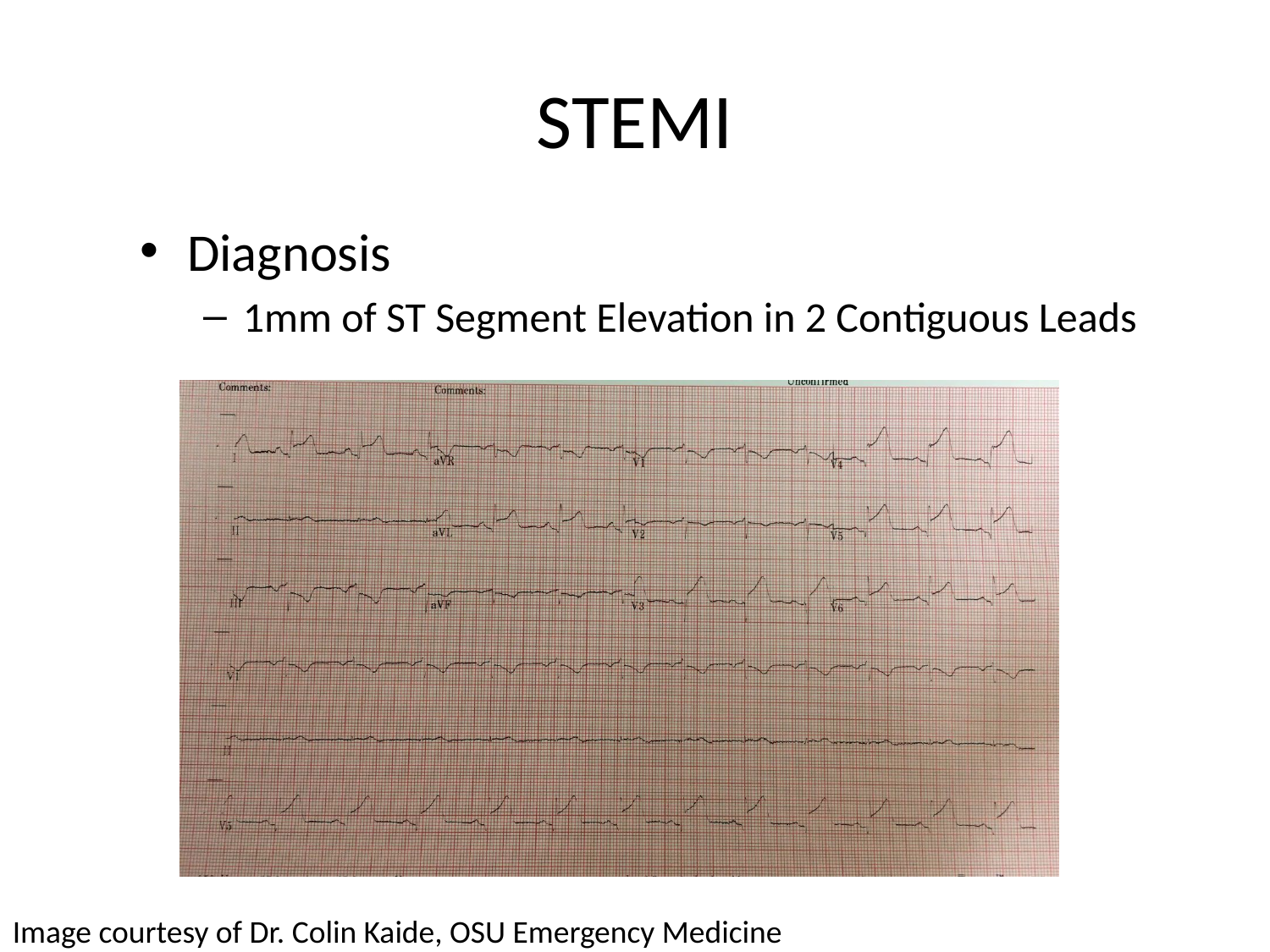

# STEMI
Diagnosis
1mm of ST Segment Elevation in 2 Contiguous Leads
Image courtesy of Dr. Colin Kaide, OSU Emergency Medicine

## Slide 5
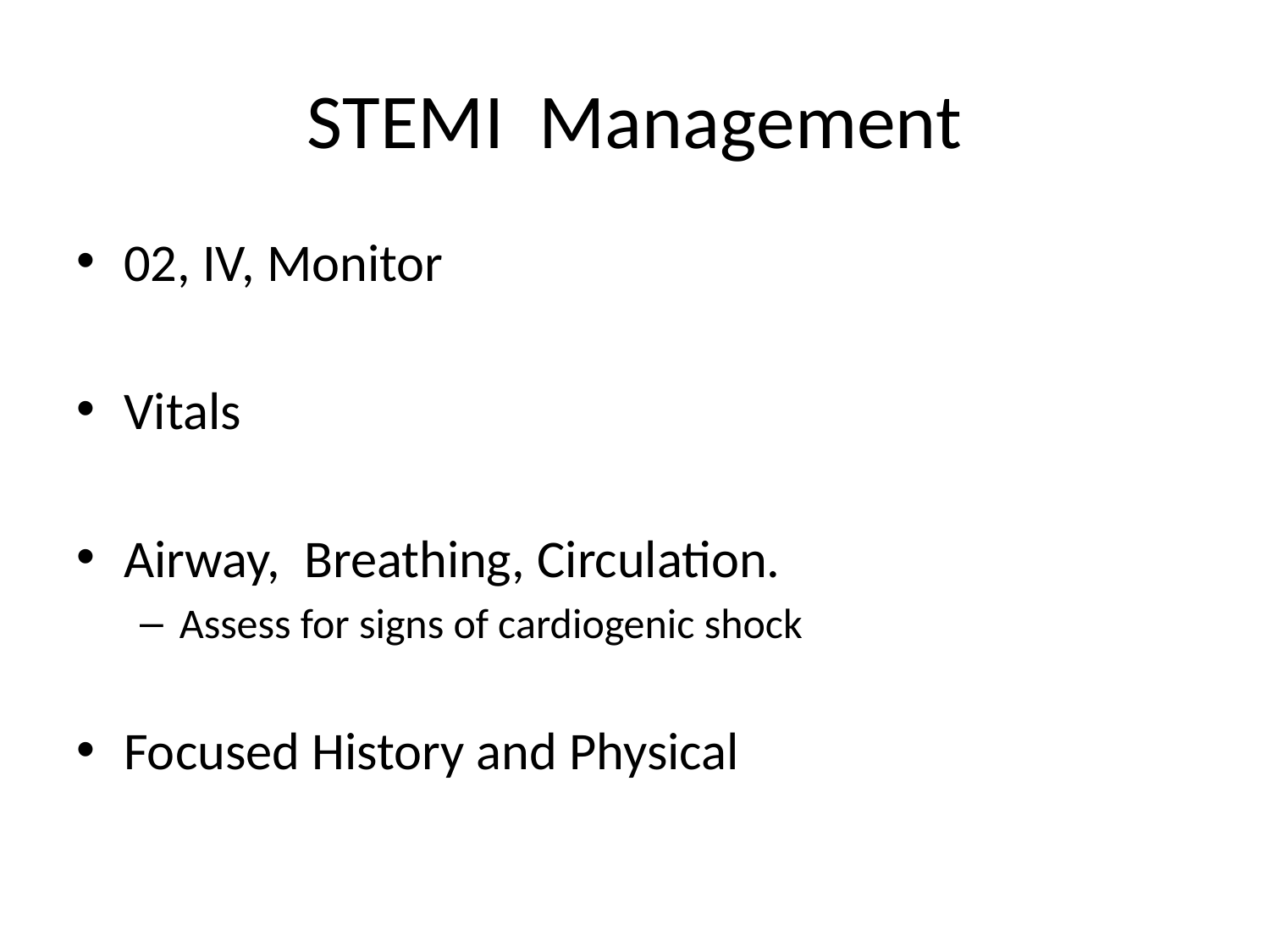

# STEMI Management
02, IV, Monitor
Vitals
Airway, Breathing, Circulation.
Assess for signs of cardiogenic shock
Focused History and Physical

## Slide 6
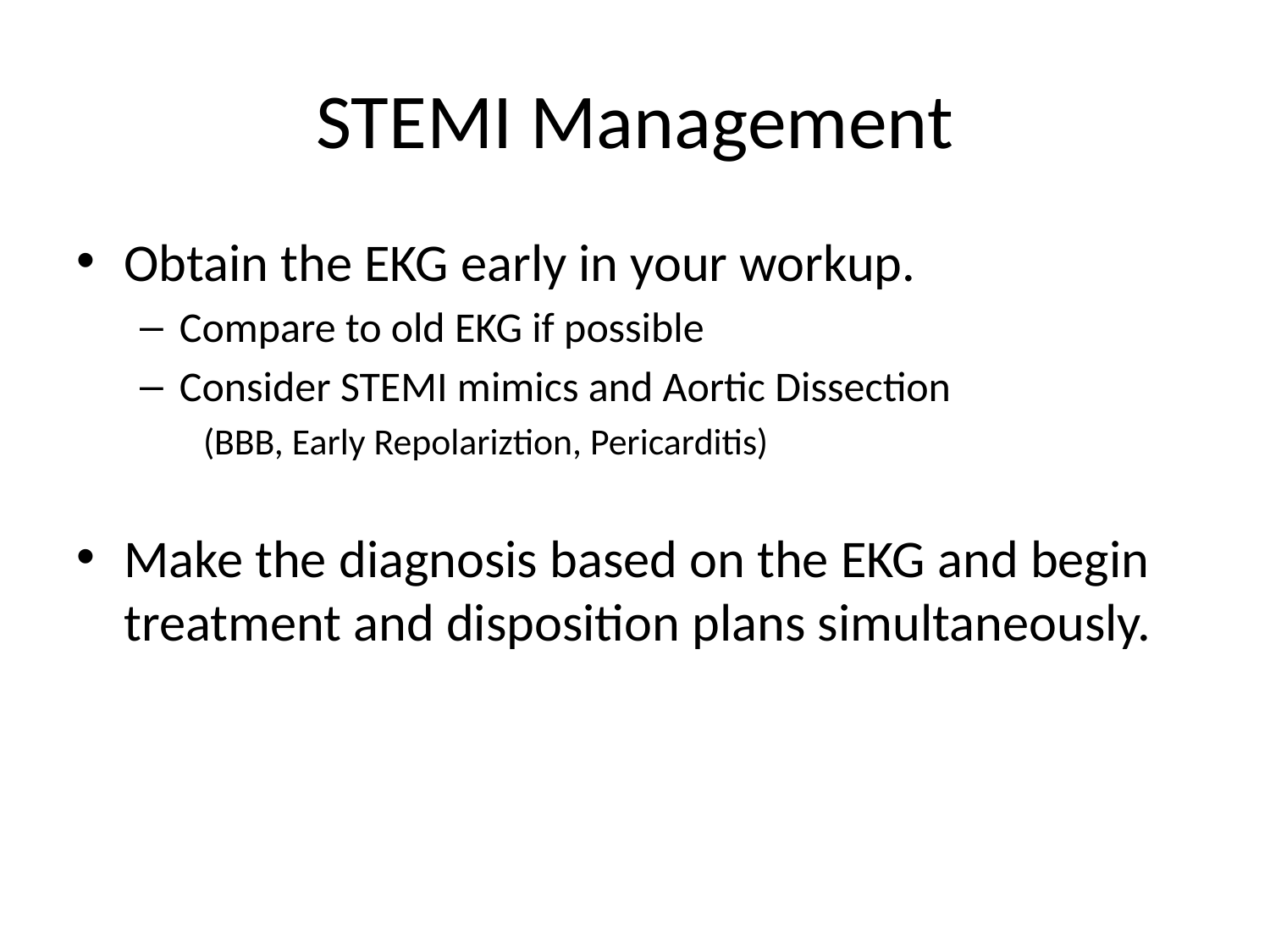

# STEMI Management
Obtain the EKG early in your workup.
Compare to old EKG if possible
Consider STEMI mimics and Aortic Dissection
(BBB, Early Repolariztion, Pericarditis)
Make the diagnosis based on the EKG and begin treatment and disposition plans simultaneously.

## Slide 7
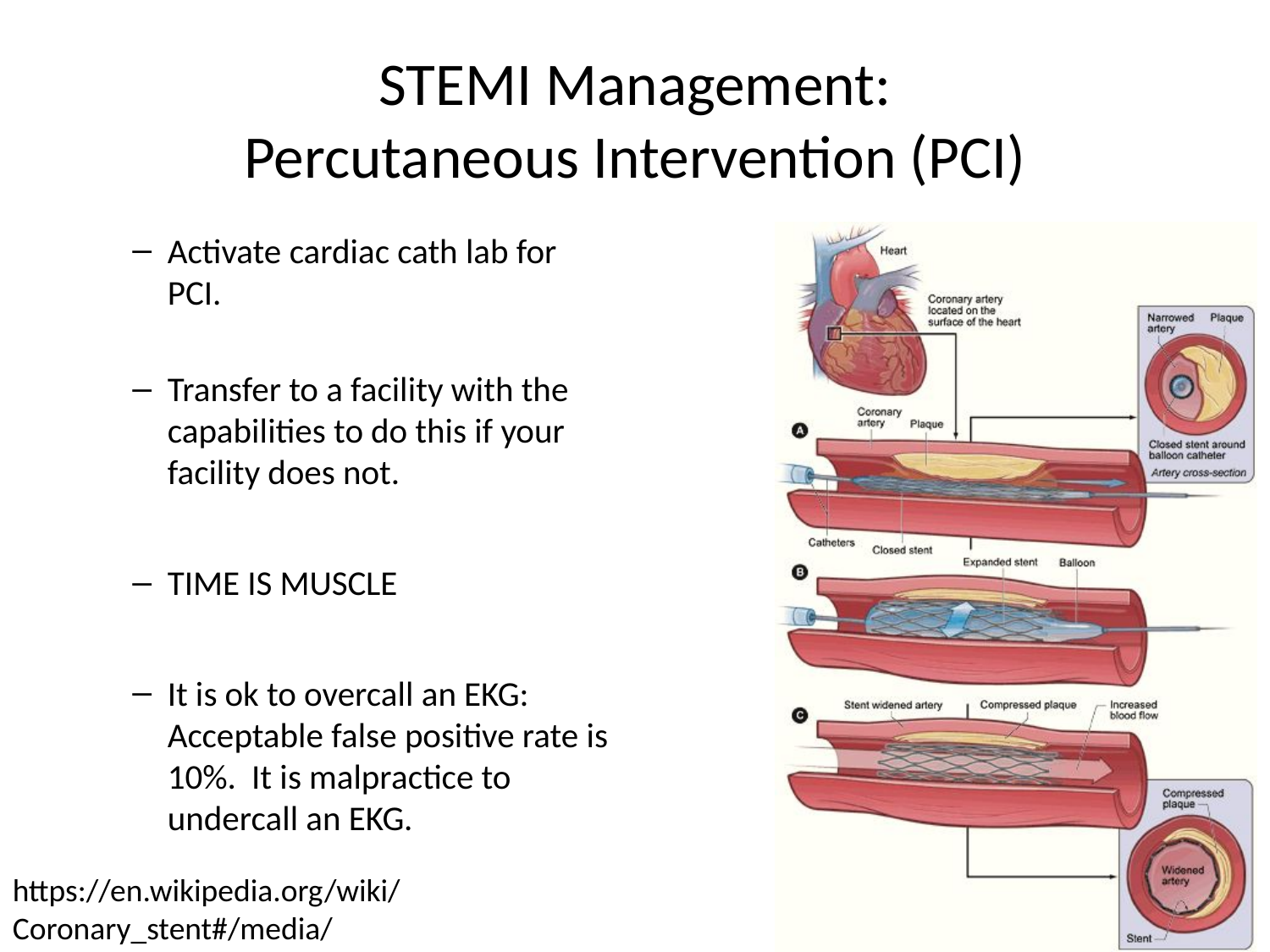

# STEMI Management: Percutaneous Intervention (PCI)
Activate cardiac cath lab for PCI.
Transfer to a facility with the capabilities to do this if your facility does not.
TIME IS MUSCLE
It is ok to overcall an EKG: Acceptable false positive rate is 10%. It is malpractice to undercall an EKG.
https://en.wikipedia.org/wiki/Coronary_stent#/media/File:PTCA_stent_NIH.gif

## Slide 8
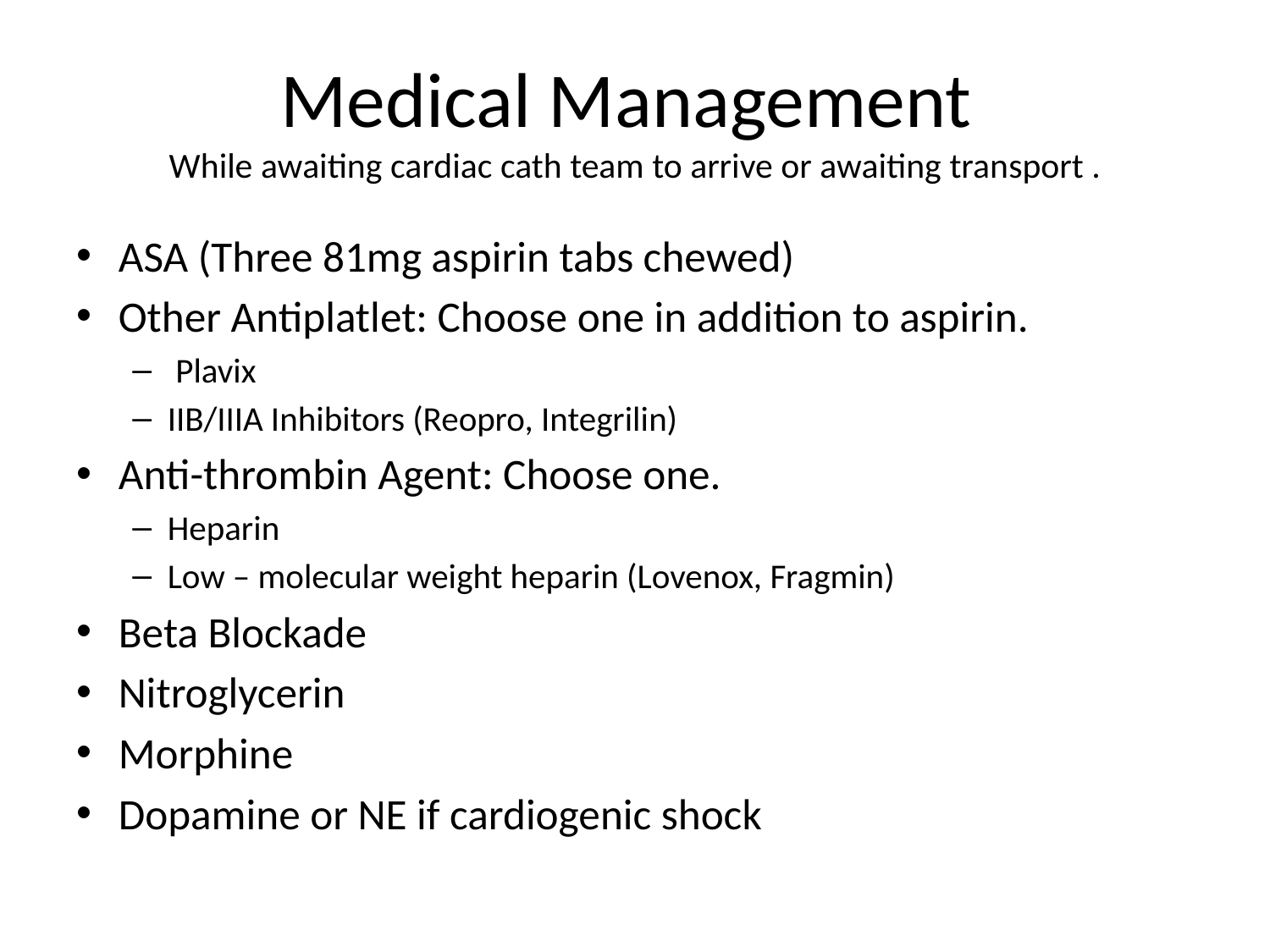

# Medical Management While awaiting cardiac cath team to arrive or awaiting transport .
ASA (Three 81mg aspirin tabs chewed)
Other Antiplatlet: Choose one in addition to aspirin.
 Plavix
IIB/IIIA Inhibitors (Reopro, Integrilin)
Anti-thrombin Agent: Choose one.
Heparin
Low – molecular weight heparin (Lovenox, Fragmin)
Beta Blockade
Nitroglycerin
Morphine
Dopamine or NE if cardiogenic shock

## Slide 9
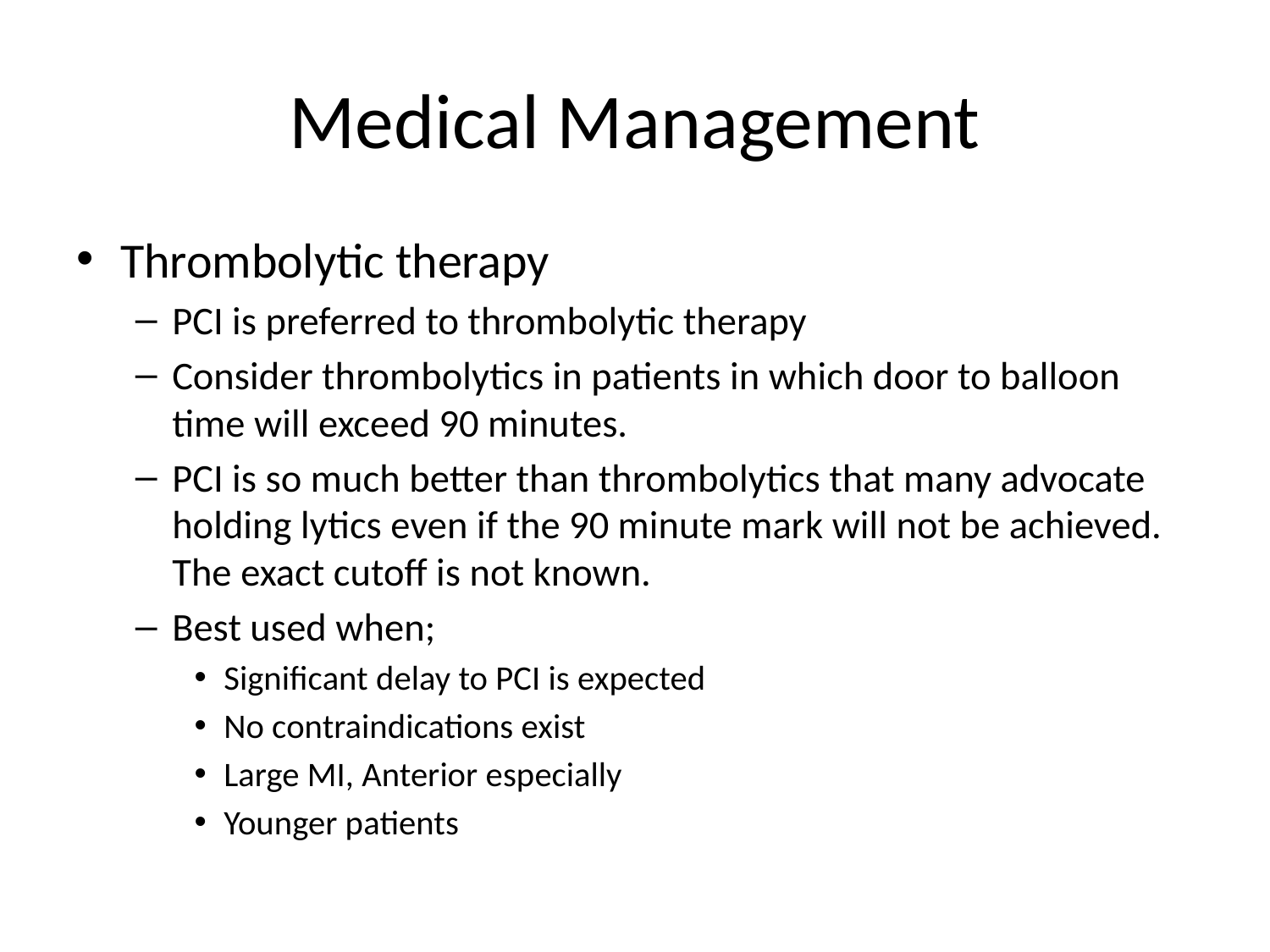

# Medical Management
Thrombolytic therapy
PCI is preferred to thrombolytic therapy
Consider thrombolytics in patients in which door to balloon time will exceed 90 minutes.
PCI is so much better than thrombolytics that many advocate holding lytics even if the 90 minute mark will not be achieved. The exact cutoff is not known.
Best used when;
Significant delay to PCI is expected
No contraindications exist
Large MI, Anterior especially
Younger patients

## Slide 10
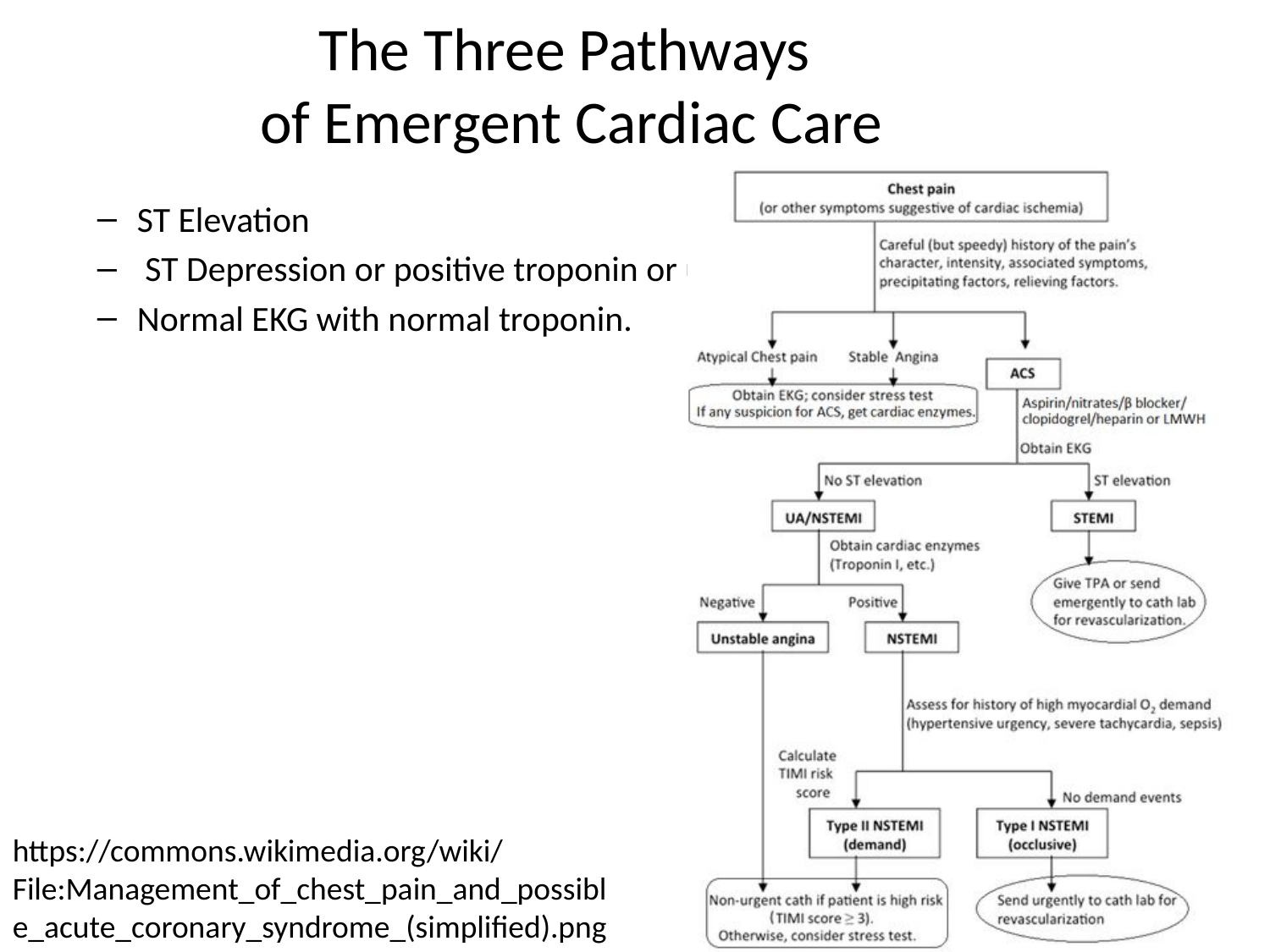

# The Three Pathways of Emergent Cardiac Care
ST Elevation
 ST Depression or positive troponin or unstable angina
Normal EKG with normal troponin.
https://commons.wikimedia.org/wiki/File:Management_of_chest_pain_and_possible_acute_coronary_syndrome_(simplified).png

## Slide 11
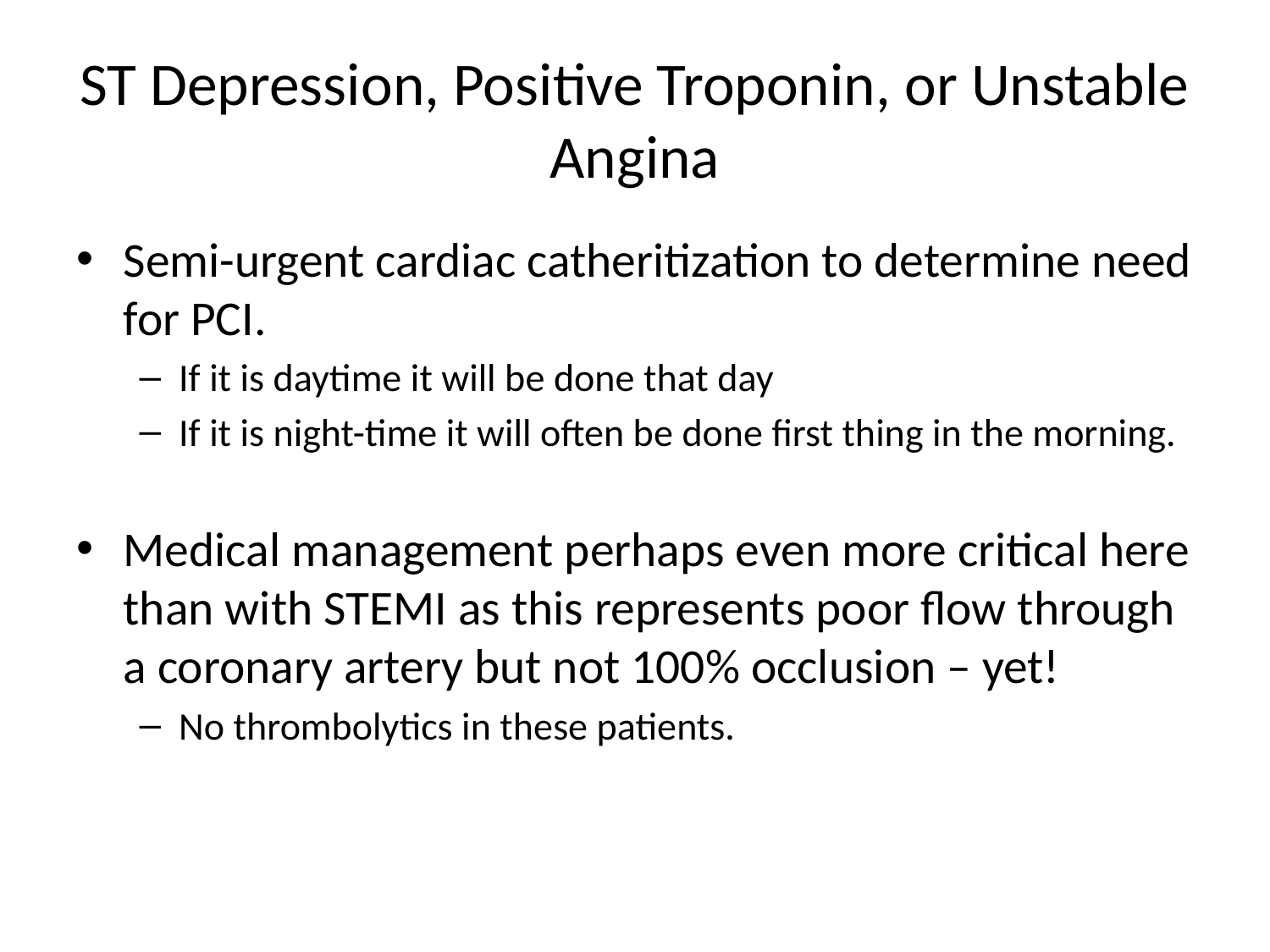

# ST Depression, Positive Troponin, or Unstable Angina
Semi-urgent cardiac catheritization to determine need for PCI.
If it is daytime it will be done that day
If it is night-time it will often be done first thing in the morning.
Medical management perhaps even more critical here than with STEMI as this represents poor flow through a coronary artery but not 100% occlusion – yet!
No thrombolytics in these patients.

## Slide 12
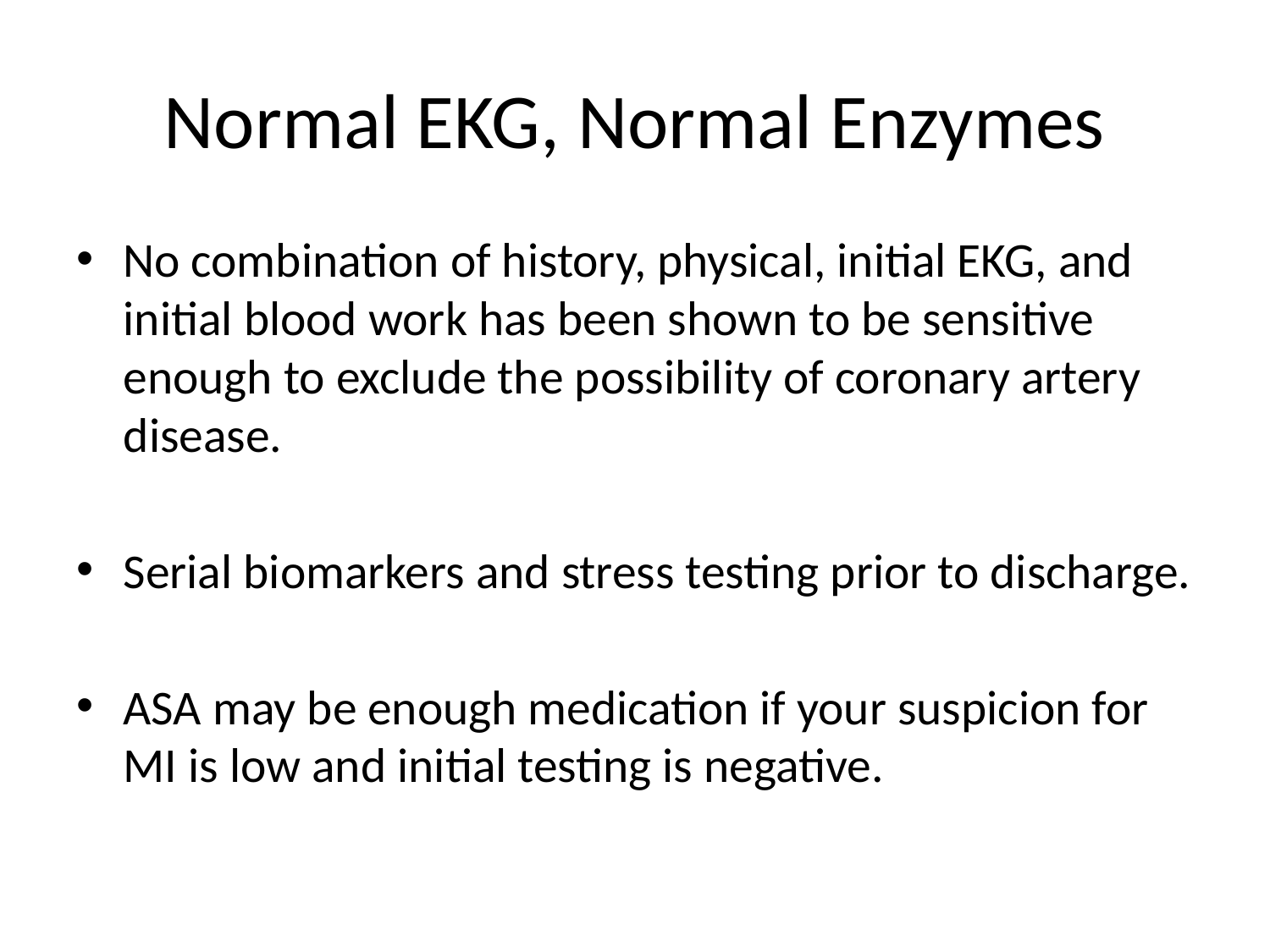

# Normal EKG, Normal Enzymes
No combination of history, physical, initial EKG, and initial blood work has been shown to be sensitive enough to exclude the possibility of coronary artery disease.
Serial biomarkers and stress testing prior to discharge.
ASA may be enough medication if your suspicion for MI is low and initial testing is negative.

## Slide 13
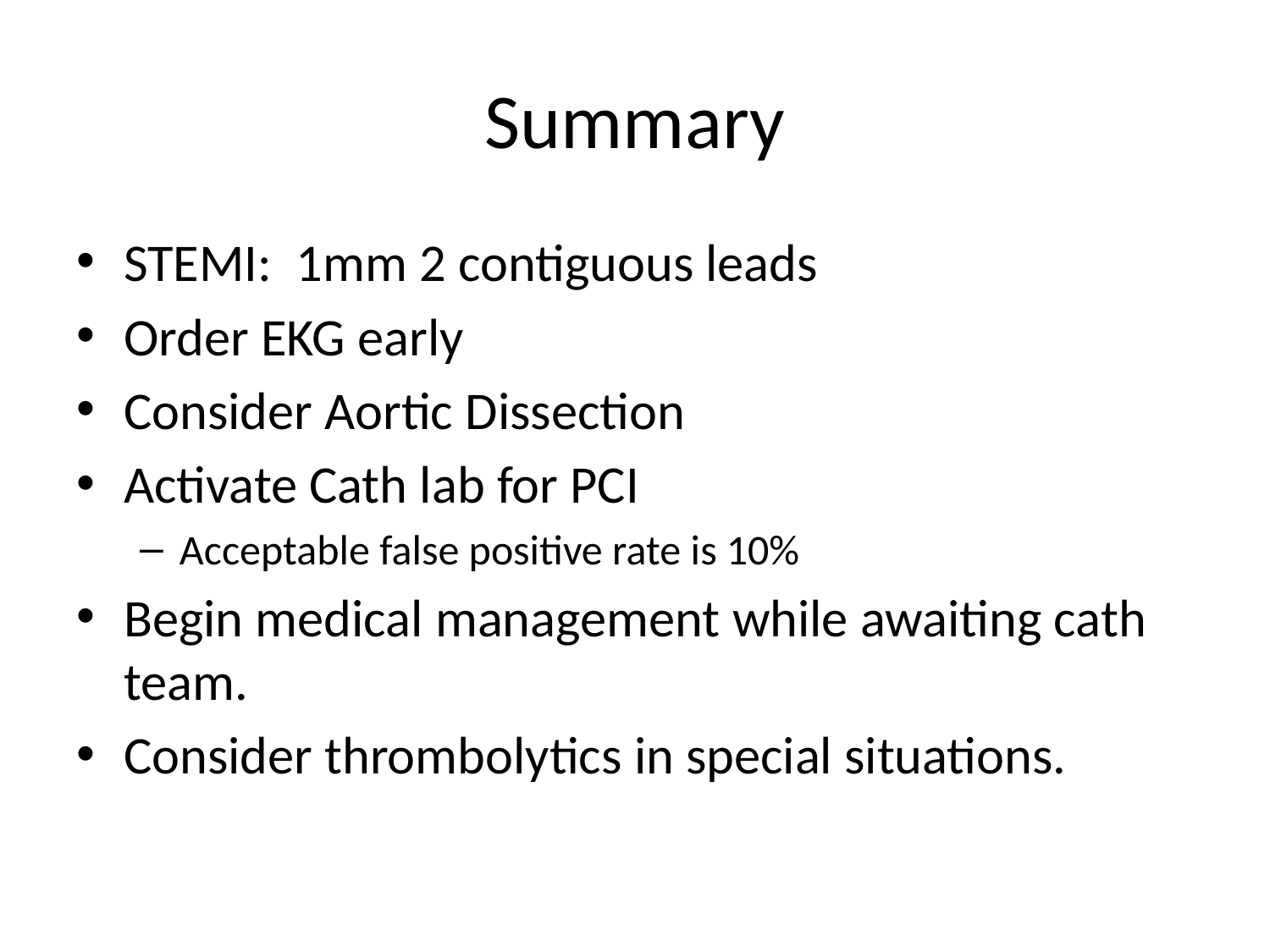

# Summary
STEMI: 1mm 2 contiguous leads
Order EKG early
Consider Aortic Dissection
Activate Cath lab for PCI
Acceptable false positive rate is 10%
Begin medical management while awaiting cath team.
Consider thrombolytics in special situations.
