# Supplementary material for: Entrustable Professional Activity 10: Case Simulation and Assessment—STEMI With Cardiac Arrest
Source: MedEdPORTAL. 2016 Dec 23;12:10517. doi: 10.15766/mep_2374-8265.10517 (PMC6440413; doi:10.15766/mep_2374-8265.10517)
Supplement: Supplementary file 1 — A. Simulation Case.docx B. Visual Stimuli.docx C. Case Assessment Rubric.docx D. STEMI Management Presentation.pptx [file mep-12-10517-s001.zip › _Educational Summary Report_10517.pdf]

MedEdPORTAL®
The Journal of Teaching and Learning Resources

Original Publication OPEN ACCESS

Entrustable Professional Activity 10: Case Simulation and Assessment—STEMI With Cardiac Arrest

Nicholas Edward Kman, MD*, Laura Thompson, MD, Jamie Hess, MD, Aaron Dora-Laskey, MD, Harsh Sule, MD, Tiffany Moadel, MD, Lalena Yarris, MD
*Corresponding author: nicholas.kman@osumc.edu

Abstract

Introduction: Entrustable professional activities (EPAs) are units of professional practice defined as tasks or responsibilities that trainees are entrusted to perform unsupervised. AAMC Core EPA 10 is defined as the ability to "recognize a patient who requires emergent care and initiate evaluation and management." We designed a simulation scenario to elicit EPA 10–related behaviors for learner assessment to guide entrustment decisions. **Methods:** This case presents a 61-year-old male with a complaint of feeling ill. The students need to diagnose an ST segment elevation myocardial infarction that leads to a pulseless ventricular tachycardia arrest. A simulation manikin is used, and students are assessed using a checklist. The tool is a set of critical actions that were proposed by a group of content experts, based on the following EPA 10 functions: recognizing unstable vital signs, asking for help, and determining appropriate disposition. In addition to case-specific behavioral items, an overall entrustment item was added to inform the entrustment decision. **Results:** This case was implemented in a mandatory fourth-year clerkship for 7 years prior to its adaptation for entrustment on EPA 10. In recent experience from one institution, about 14% of students failed to meet entrustment. Students rated the experience as valuable (average 5.0, on a 5-point Likert scale) and thought that it would change their performance in a clinical setting (average 4.95, on a 5-point Likert scale). **Discussion:** Faculty raters noted challenges regarding entrustment based on a single simulation and the implications that team role (supporting role vs. leader role) has on entrustment.

Keywords

Entrustable Professional Activity, Emergency Medicine, Patient Simulation

Citation: Kman NE, Thompson L, Hess J, et al. Entrustable Professional Activity 10: case simulation and assessment—STEMI with cardiac arrest. *MedEdPORTAL*. 2016;12:10517. https://doi.org/10.15766/mep_2374-8265.10517

Copyright: © 2016 Kman et al. This is an open-access article distributed under the terms of the Creative Commons Attribution license.

Appendices

A. Simulation Case.docx
B. Visual Stimuli.docx
C. Case Assessment Rubric .docx
D. STEMI Management Presentation.pptx

All appendices are peer reviewed as integral parts of the Original Publication.

Educational Objectives

By the end of this simulation, learners will be able to:

1. Recognize normal vital signs and variations that might be expected based on patient- and disease-specific factors.
2. Recognize severity of a patient's illness and indications for escalating care.
3. Identify potential underlying etiologies of the patient's decompensation.
4. Apply basic and advanced life support as indicated.
5. Start an initial care plan for the decompensating patient.
6. Engage team members required for immediate response, continued decision making, and necessary follow-up to optimize patient outcomes.
7. Initiate a code response and participate as a team member.
8. Communicate the situation to responding team members.

Introduction

AAMC Core Entrustable Professional Activity (EPA) 10 outlines the physician's ability to promptly recognize a patient who requires urgent or emergent care, initiate evaluation and management, and seek help and is an essential trait for medical students to develop. This EPA often calls for simultaneously recognizing need

and initiating a call for assistance. The current resource was created by educators at multiple institutions, some of which are participating in the EPA pilot study. This case and simulation were developed based on the AAMC Core Entrustable Professional Activities for Entering Residency: Curriculum Developers' Guide[1] and aim to assess this skill in senior medical students.

At the time of this project, there were no published tools that directly assessed students' abilities to perform the tasks of EPA 10. In the interim, a related simulation case of ours, "Entrustable Professional Activity 10: Recognizing the Acutely Ill Patient—A Delirium Simulated Case for Students in Emergency Medicine,"[2] has been published on MedEdPORTAL.

A variation on this simulation case has been used for several years at one institution. The target audience of the simulation is medical student educators, with the participants being fourth-year medical students. Prerequisites include completion of the core clerkships of the third year of medical school.[3]

Methods

The goal of simulation is to improve the speed, skill, and acumen of learners in a setting safe for errors so that when learners encounter a critical patient, they are able to perform at a higher level.[4] We chose simulation as a method to assess EPA 10 as it is difficult to ensure that each student will get an opportunity to treat a patient with an emergent need and initiate treatment.[5] This simulation is used in a mandatory fourth-year emergency medicine clerkship for as many as 245 students per year.

The simulation case (Appendix A) presents a 61-year-old male who has fallen ill. The students need to diagnose an ST segment elevation myocardial infarction (STEMI) that leads to a pulseless ventricular tachycardia arrest. A document with visual stimuli (Appendix B), including X-rays, CT scans, EKGs, and lab values, is also included for learner use.

At our institution, we run four different cases with our students in teams of four students per case. Eventually, each student gets to be the team leader once during the session. Students are prepped prior to the case for 5 minutes and get about 15 minutes to run the case and 10 minutes to debrief. We run four cases in 2 hours (30 minutes per case). The cases are run on the second to last day of the clerkship as a part of a 2-day assessment period (students take the National Board of Medical Examiners emergency medicine exam on the last day). The only preparation given to students is the direction to review advanced cardiovascular life support (ACLS) prior to the simulation.

Equipment/Environment

Simulations are conducted in a replica of an emergency department resuscitation bay in our Clinical Skills Education and Assessment Center. The Laerdal SimMan manikin is used for all simulated cases. The manikin is programmed and controlled by a simulation technician and a faculty facilitator seated in the control room behind a one-way glass window. Specific items that can be used or placed on the cart during the simulation include the following:

- Manikin setup: iSTAN high-fidelity monitor or comparable.
- Props: See Appendix B for STEMI ECG, normal EKG, chest X-ray, and ventricular tachycardia EKG.
- Airway equipment (nasal cannula, non-rebreather, bag-valve mask, bilevel positive airway pressure, blades, endotracheal tubes, end-tidal CO_2, and stethoscope).
- Basic monitor, pulse oximeter, and how to attach blood pressure cuff.
- ACLS equipment (monitor defibrillator, medication list, and ACLS algorithms).
- Ultrasound for trauma, cardiac arrest, and pulseless electrical activity.
- Medication list: aspirin, plavix, heparin/low-molecular-weight heparin, Gp2b3a inhibitor, consideration of nitroglycerin but hold for hypotension, and morphine. Vasoactive medications per ACLS guidelines (epinephrine or vasopressin, amiodarone) at appropriate doses and intervals.

Personnel

Students participate in this simulation in teams of four, with one student being the code leader. Additional roles for the remaining students are airway (head of bed) and two circulators (running monitor defibrillator and performing chest compressions). At our institution, this is one of four cases used to assess EPA 10. All

students in the group participate in all four cases during the simulation session. Each student is designated team leader for one of the four cases and is responsible for assigning tasks to the other team members, such as performing procedures, obtaining consent, or communicating with family and consultants. The team-leader role rotates for each case so that each student in the group is the team leader for at least one case. Faculty evaluates students during the case in which they are the team leader.

This scenario requires one simulationist to run the manikin and one faculty member to assess the students and help with the simulation. These personnel are behind the glass and may be used to provide cues to the students, deliver lab values or imaging, and answer questions (if appropriate) or redirect them if needed. For standardization and interrater reliability, a team of three simulationists and five faculty is responsible for the delivery of all the simulations.

The patient's voice is provided by the faculty facilitator or simulation technician through a microphone embedded in the SimMan. The roles of family members, consultants, and other medical staff are voiced by the faculty facilitator through a telephone or overhead speaker.

Assessment

An expert panel of four education faculty (three emergency medicine and one anesthesia) was tasked with creating the checklist assessment of critical actions (Appendix C). The AAMC EPA Curriculum Developers Guide was used to identify expected behaviors for a learner who could be entrusted to recognize a patient requiring urgent and emergent care, initiate evaluation and management, and seek help within the clinical contexts assessed.[1]

Three types of checklist items for assessing student performance on the simulated case were developed by the panel. First, a set of three universal critical actions was identified: "Interprets vital signs and recognizes severity of a patient's illness and indications for escalating care," "Engages team members in immediate response," and finally, "Reassesses patient's condition, continues decision making and requisite follow-up."

Universal checklist items were supplemented with three to five case-specific critical actions related to the identification of potential underlying etiologies of the patient's decompensation, the start of initial care plans for the decompensating patient, and application of basic and advanced life support.

The checklist items were reviewed by experts in critical care, simulation development, and assessment. The final set of critical actions represents consensus among the expert panel. The checklist instrument for the case simulation also includes a global rating of entrustment.

Debriefing

Each simulation lasts approximately 15-20 minutes and is followed by a 10- to 15-minute debriefing session. The first few minutes of the debriefing session are devoted to self-reflection by the participants and feedback from the faculty facilitator. The remainder of the debriefing session consists of a brief, focused review of the case objectives. The final portion is a review of STEMI management using the PowerPoint presentation (Appendix D).[6]

Results

One hundred fourteen medical students, or 62% of the total class (114 out of 185), participated in the EPA 10 assessment between June and December of 2015. Overall, students met the criteria for universal critical actions: specifically, recognizing unstable vital signs (97%), asking for help from ancillary and medical staff (93%), and appropriate disposition (92%). Faculty raters (five total for the year) reached a judgment of entrustment for 86% of students at the end of the case. Between groups of students based on rotation, there was very little variability, and no more than four students in any cohort of 24 students were determined not to have met ad hoc entrustment (17%).

Some selected comments from the students after completion of the simulation case include the following:

- "Simulation at the end of the EM rotation was very useful, particularly because of the feedback portion—this helped pinpoint what went well and what needed improvement."

MedEdPORTAL®
The Journal of Teaching and Learning Resources

- "The emergency medicine simulation and debriefing was excellent."
- "I loved the simulations that were part of the EM block. It was a great opportunity to put yourself to the test and find out what you would do when a patient was decompensating in front of you. I wish the simulations would get incorporated into more blocks throughout third and fourth year because they are the most efficient way to consolidate medical knowledge and identify gaps in your knowledge that you didn't even realize you didn't know."

Discussion

EPAs represent the foundation of modern medical training. Measurement of these functions ensures a trainee's ability to perform to accepted standards of care. Medical schools and residency programs have a responsibility to the public to assure that their graduates have been assessed for entrustment of these activities prior to unsupervised practice.[7] To meet this responsibility, medical educators must integrate high-quality, formal EPA assessments into their training programs.

EPA 10 is particularly important because it requires the medical trainee to recognize an unstable patient who requires lifesaving emergent care.[8] Multiple teaching methods have been employed to familiarize and prepare trainees for these clinical situations, including high-fidelity simulations (HFSs). HFSs have been firmly established by teaching programs and embraced by trainees for the presentation of realistic scenarios that illustrate pathophysiology and challenge the learner to apply medical knowledge and clinical acumen. In this pilot project, we addressed the issue of assessing the trainee for entrustment of EPA 10. We developed and piloted a checklist instrument for establishing trainee entrustment during HFSs involving unstable patients in an acute care setting. We evaluated the EPA 10 assessment by conducting an expert panel review (including emergency, critical care, and simulation medicine) of each case for content and critical actions to establish entrustment.

During this pilot, we confronted several limitations. First, we were unable to completely isolate an individual student's performance from the performance of the team. However, trained evaluators were asked to note situations in which the team leader was being carried by the team. A second limitation was the product of the manner in which we conducted our assessments. Since teams of four students rotated through the team-leader position (i.e., the position that was evaluated), there could have been a cumulative practice benefit for the students who were last to perform.

Other considerations and limitations on generalizability may include equipment availability, time investment of faculty and support staff, and transference from simulation to actual patient care environments. High-fidelity human physiology simulation equipment and qualified technical support staff require significant institutional monetary investment. For standardization and interrater reliability, a team of three simulationists and five faculty was responsible for the delivery of all the simulations. From this group, for each student assessment, we used one trained physician faculty rater and a trained simulator specialist. Each assessment lasted up to 35 minutes per student. Training nonphysician raters could be considered for cost savings. Simultaneous assessments of multiple students with different roles or with responsibility for different critical actions could also be considered.

Nicholas Edward Kman, MD: Associate Professor of Emergency Medicine, Ohio State University College of Medicine

Laura Thompson, MD: Assistant Professor of Emergency Medicine, Ohio State University College of Medicine

Jamie Hess, MD: Assistant Professor of Emergency Medicine, University of Wisconsin School of Medicine and Public Health

Aaron Dora-Laskey, MD: Clinical Lecturer, University of Michigan Medical School

Harsh Sule, MD: Residency Program Director, Rutgers Biomedical and Health Sciences

Tiffany Moadel, MD: Instructor in Emergency Medicine and Director of Medical Student Simulation, Yale School of Medicine

Lalena Yarris, MD: Associate Professor, Department of Emergency Medicine, Oregon Health & Science University School of Medicine

Disclosures
None to report.

Funding/Support
None to report.

Ethical Approval
This publication contains data obtained from human subjects and received ethical approval.

References

1. Core entrustable professional activities for entering residency: curriculum developers' guide. Association of American Medical Colleges Web site. https://members.aamc.org/eweb/upload/core%20EPA%20Curriculum%20Dev%20Guide.pdf. Accessed January 28, 2016.
2. Dora-Laskey A, Sule H, Moadel T, et al. Entrustable Professional Activity 10: recognizing the acutely ill patient—a delirium simulated case for students in emergency medicine. *MedEdPORTAL Publications*. 2016;12:10512. https://doi.org/10.15766/mep_2374-8265.10512
3. Thompson L, Leung C, Green B, et al. Assessment of EPA-10 in a fourth year emergency medicine clerkship. MedEdPORTAL iCollaborative Web site. https://www.mededportal.org/icollaborative/resource/4199. Published January 25, 2016. Accessed January 28, 2016.
4. Morgenstern BZ, ed. *Guidebook for Clerkship Directors.* 4th ed. North Syracuse, NY: Gegensatz Press; 2012.
5. Wayne DB, Siddall VJ, Butter J, et al. A longitudinal study of internal medicine residents' retention of advanced cardiac life support skills. *Acad Med*. 2006;81(10)(suppl):S9-S12. https://doi.org/10.1097/00001888-200610001-00004
6. Patwari R. Acute coronary syndromes. CDEM Curriculum Web site. http://cdemcurriculum.com/cardiovascular/acute-coronary-syndromes/. Updated 2008. Accessed February 23, 2016.
7. Englander R, Flynn T, Call S, et al. Toward defining the foundation of the MD degree: core entrustable professional activities for entering residency. *Acad Med*. 2016;91(10):1352-1358. https://doi.org/10.1097/ACM.0000000000001204
8. McEvoy MD, Dewaay DJ, Vanderbilt A, et al. Are fourth-year medical students as prepared to manage unstable patients as they are to manage stable patients? *Acad Med*. 2014;89(4):618-624. https://doi.org/10.1097/ACM.0000000000000192

Received: May 4, 2016 | **Accepted:** December 2, 2016 | **Published:** December 23, 2016
