# Supplementary material for: Entrustable Professional Activity 10: Case Simulation and Assessment—STEMI With Cardiac Arrest
Source: MedEdPORTAL. 2016 Dec 23;12:10517. doi: 10.15766/mep_2374-8265.10517 (PMC6440413; doi:10.15766/mep_2374-8265.10517)

| Appendix A: MedEdPORTAL Simulation Case Template  SIMULATION CASE TITLE: EPA 10: Case Simulation –STEMI with Cardiac Arrest  AUTHORS: Kman NE, Thompson L, Hess J, Dora-Laskey A, Sule H, Moadel T, Yarris L. | |
| --- | --- |
| PATIENT NAME: Stan Smith  PATIENT AGE: 61  CHIEF COMPLAINT: “feeling ill” with chest heaviness, anxious, nauseated | |
|  | |
| Brief narrative description of case  *Include the presenting patient chief complaint and overall learner goals for this case* | The patient presents with sudden onset chest heaviness radiating to his jaw, anxiety, diaphoresis and nausea. His symptoms are progressively worsening since onset 1 hour ago and arrives to the ED ill appearing  The goals of this case are to enable students to show mastery of EPA 10, with the goal of “recognizing a patient requiring urgent or emergent medical care and initiate evaluation and management.” |
| Primary Learning Objectives  *What should the learners gain in terms of knowledge and skill from this case? Use action verbs and utilize Bloom’s Taxonomy as a conceptual guide* | Primary learning objectives mirror EPA-10 learning objectives   1. Recognize a patient who requires emergent care 2. Identify normal and abnormal vital signs 3. Recognize the severity of the patient illness and risk of decompensation 4. Demonstrate teamwork skills in managing a critical patient 5. Initiate care during a code and appropriately engage team members |
| Critical Actions  *List which steps the participants should take to successfully manage the simulated patient. These should be listed as concrete actions that are distinct from the overall learning objectives of the case.* | 1. Identify STEMI on EKG 2. Give antiplatelet agents (ASA, plavix) 3. Give anticoagulation (heparin/low molecular weight heparin, gp2b3a inhibitors) 4. Arrange for emergent cardiac catheterization vs Thrombolytic Treatment (one or the other) 5. Administer high quality chest compressions 6. Manage Pulseless Ventricular Tachycardia (early defibrillation, drug-shock-antiarrhythmic) |
| Learner Preparation  *What information should the learners be given prior to initiation of the case?* | Learners should be given an introduction the simulation environment and capabilities of the sim mannequin (what physical exam findings can they rely on vs when to ask questions) |

| Initial Presentation | | | |
| --- | --- | --- | --- |
| Initial vital signs | - - 1. Heart rate 95     2. Blood pressure 100/65     3. Respiratory rate 22     4. Pulse ox 95%     5. Temperature 98.8. | | |
| Overall Appearance  *What do learners see when they first enter the room?* | 61yo Male appearing uncomfortable, slightly tachypneic and obviously diaphoretic. This patient is sitting on the gurney and is awake and alert. | | |
| Actors and roles in the room at case start  *Who is present at the beginning and what is their role? Who may play them?* | In addition to the patient, the simulation technologist or Faculty member can verbalize handoff from the EMS team. This is a brief role and can be done by anyone. | | |
| HPI  *Please specify what info here and below must be asked vs what is volunteered by patient or other participants* | The patient is a 61 year old male with complaint of “feeling ill” brought by BLS transport. Medics tell you “we are a BLS ambulance and didn’t give him anything yet”. The patient complains of feeling very ill beginning one hour ago. He was sitting at a college football game and eating nachos when he began feeling anxious, sweaty, and nauseated. This was associated with chest heaviness radiating to the jaw and mild shortness of breath that have been getting progressively worse since onset. He denies any history of similar symptoms. | | |
| Past Medical/Surgical History | Medications | Allergies | Family History |
| Hypertension, hyperlipidemia, diabetes | Insulin sliding scale and HCTZ | NKDA | Father and brother with heart problems/stents |
| Physical Examination | | | |
| General | Uncomfortable appearing, profusely diaphoretic, slightly tachypneic | | |
| HEENT | Normal | | |
| Neck | No JVD | | |
| Lungs | mild tachypnea, CTA bilaterally | | |
| Cardiovascular | RRR, no murmur, pulses +2 equal bilateral upper and lower extremities | | |
| Abdomen | soft, NT, no masses | | |
| Neurological | Alert and oriented, grossly normal neuro exam | | |
| Skin | well-perfused, trace edema | | |
| GU | Normal | | |
| Psychiatric | Anxious | | |

| Instructor Notes - Changes and CASE Branch Points  *This section should be a list with detailed description of each step than may happen during the case. If medications are given, what is the response? Do changes occur at certain time points? Should the nurse or other participant prompt the learners at given points? Should new actors or participants enter, and when? Are there specific things the patient will say or do at given times? There are a few examples given, but it is expected that most cases will have many more changes and potential branch points..* | | |
| --- | --- | --- |
| Intervention / Time point | Change in Case | Additional Information |
| After initial assessment, Students request EKG | Recognize STEMI, order meds and | If no EKG is requested, RN will prompt students by asking if they are concerned about his chest pain. |
| Students should ask to call cardiology | cardiologist should say, whether or not they gave lytics… “I want to take this patient to the cath lab…. how are you sending this patient, air or ground.” |  |
| After plans for transportation made, patient will state “I don’t feel so good” and become unresponsive | Students should start ACLS. The students should lower the head of the bed and begin BVM and chest compressions | If students do not check for a pulse and start chest compressions, simulationist or nurse confederate (if available) will prompt, “should I call a code?” |
| Patient goes into Ventricular Tachycardia rhythm at 2 minute check | Students should debribrillate, give Epi and give Amiodarone | If students forget Amiodarone, simulationist or nurse confederate (if available) can prompt with question “are there any other drugs we should give for this arrhythmia?” |
| intubation | Only necessary if students do not recognize pulseless V Tach and initiate care consistent with ACLS protocols. |  |
| Post Cardiac Arrest Care | If the patient was intubated, students should report that therapeutic hypothermia will be induced. If the patient responded quickly to ACLS and is awake, this is not necessary. |  |

Ideal Scenario Flow

*Provide a detailed narrative description of the way this case should flow if participants perform in the ideal fashion.*

1. *Initial approach to the undifferentiated patient encounter*
   - - 1. *ABC’s, O2-IV-Monitor*
       2. *Focused history and physical examination*
       3. *EKG, Blood work, Chest Xray ordered at same time as treatment initiated.*
2. *Management of STEMI*
   - - 1. *EKG interpretation. Treatment without waiting for troponin.*
       2. *PCI vs thrombolytics*
       3. *Therapy while awaiting reperfusion*
       4. *Antiplatelet (Aspirin, Clopidogrel), anti-thrombin (Heparin, Low Molecular Weight Heparin)*
       5. *Talking to EMS, family, patient, and consultant regarding diagnosis*
3. *Management of pulseless V-tach and v-fib arrest*
   - - 1. *Defibrillation*
       2. *High quality CPR and chest compressions*
4. *Airway management*
5. *Role of post-cardiac arrest care*

Anticipated Management Mistakes

*Provide a list of management errors or difficulties that are commonly encountered when using this simulation case.*

1. *Difficulty with bedside monitors: We found when using this case with medical students that many of our learners did not know how to properly connect EKG leads to the bedside monitor. We modified our sessions to include an introduction to simulation cases that includes a tutorial for connecting patients to bedside monitoring.*
2. *Failure to recognize the STEMI: Some of our learners did not immediately recognize that the patient was having an STEMI.*
3. *Failure to recognize Pulseless Ventricular Tachycardia: Learners have to recognize V Tach and initiate the appropriate ACLS protocols.*
4. *Uncertainty about indications for thrombolysis: Many of our learners were unfamiliar with the indications for the use of revascularization in acute MI. We created specific debriefing materials to cover this information.*

Example image of a sample cart for use during the simulation case.


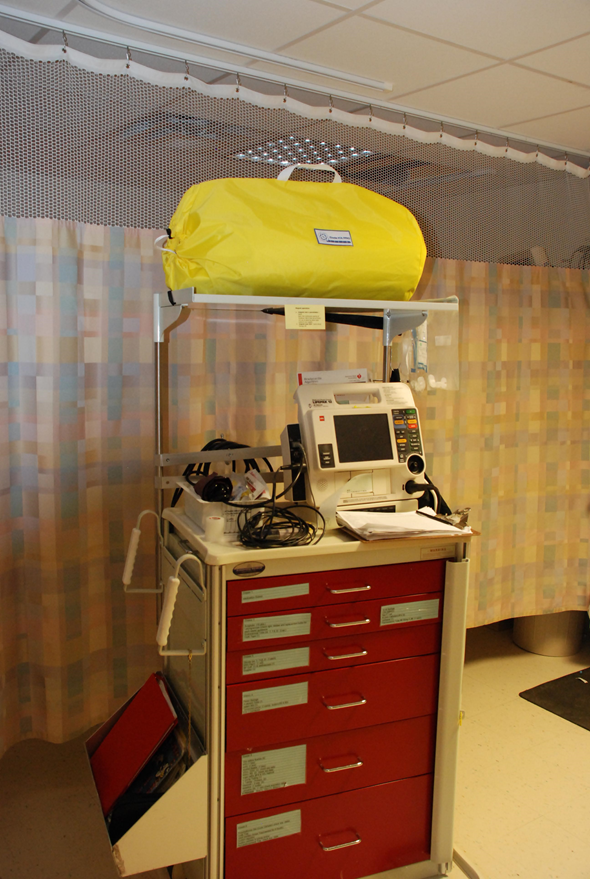

Supplement: Supplementary file 1 — A. Simulation Case.docx B. Visual Stimuli.docx C. Case Assessment Rubric.docx D. STEMI Management Presentation.pptx [file mep-12-10517-s001.zip › A. Simulation Case.docx]
