# Supplementary material for: Entrustable Professional Activity 10: Case Simulation and Assessment—STEMI With Cardiac Arrest
Source: MedEdPORTAL. 2016 Dec 23;12:10517. doi: 10.15766/mep_2374-8265.10517 (PMC6440413; doi:10.15766/mep_2374-8265.10517)
Supplement: Supplementary file 1 — A. Simulation Case.docx B. Visual Stimuli.docx C. Case Assessment Rubric.docx D. STEMI Management Presentation.pptx [file mep-12-10517-s001.zip › B. Visual Stimuli.docx]

**Appendix B**

This file should contain all visual stimuli needed to implement your case. This should include patient de-identified x-ray images, CT scans, EKGs, and laboratory values.


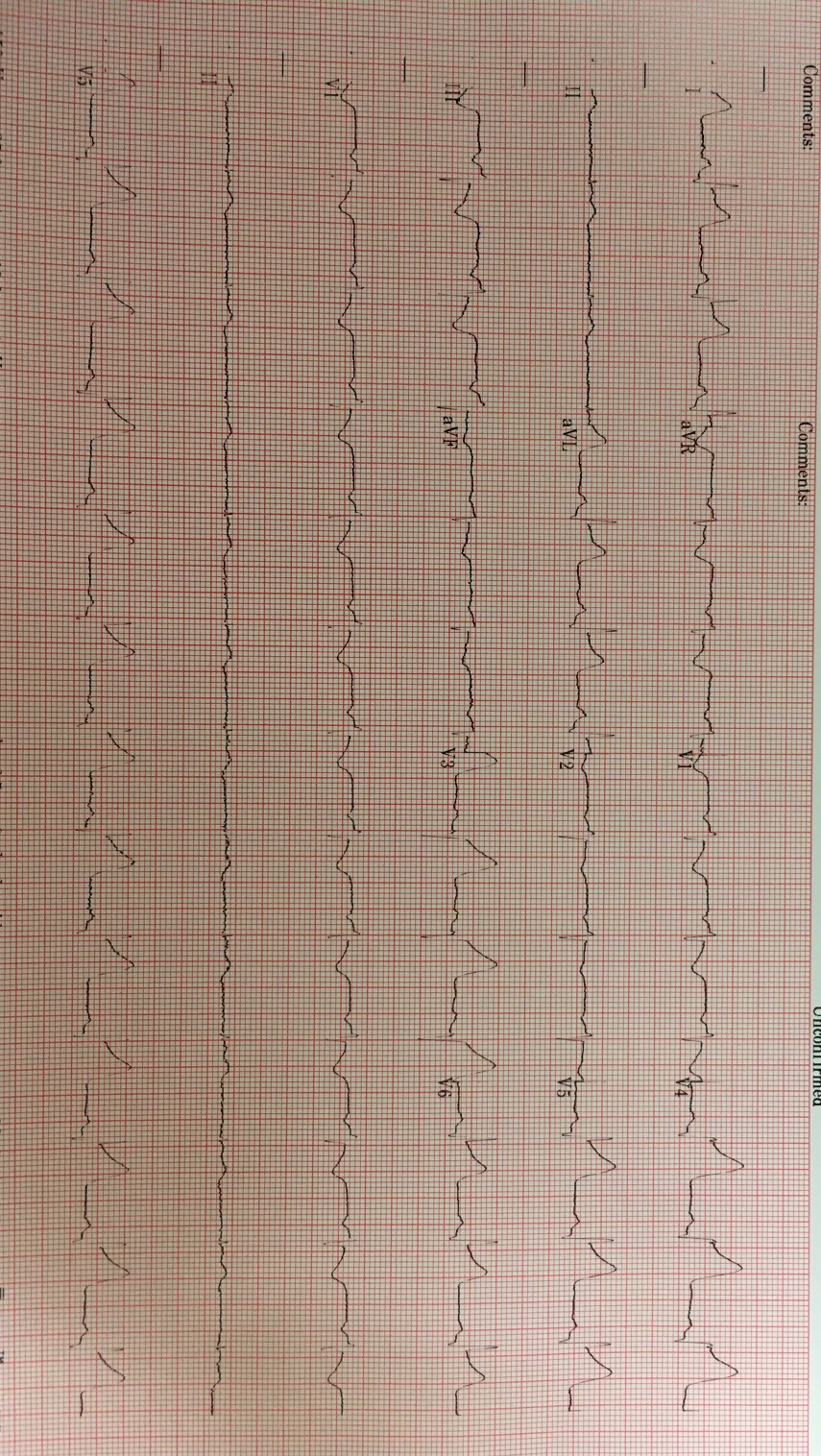


Figure 1: EKG showing lateral STEMI. Photo courtesy of Dr. Colin Kaide, OSU Emergency Medicine.


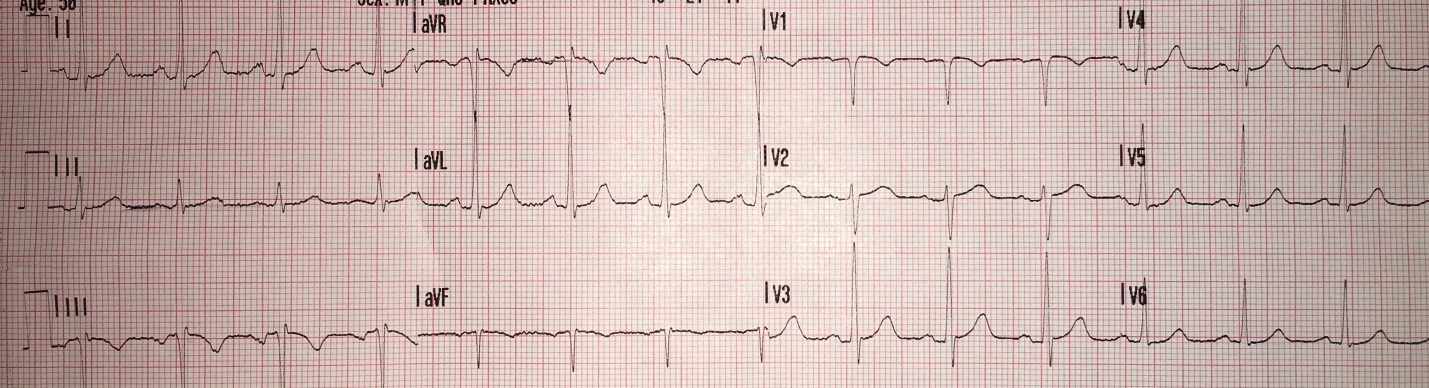


Figure 2: Normal EKG. Photo courtesy of Dr. Colin Kaide, OSU Emergency Medicine


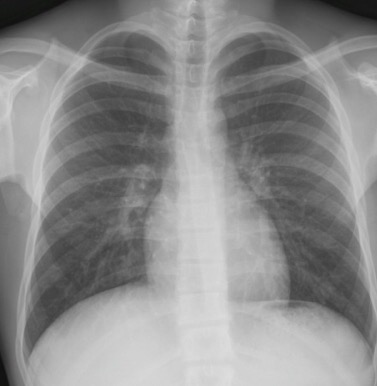


Figure 3: Chest X-Ray. Photo courtesy of Dr. Colin Kaide, OSU Emergency Medicine.


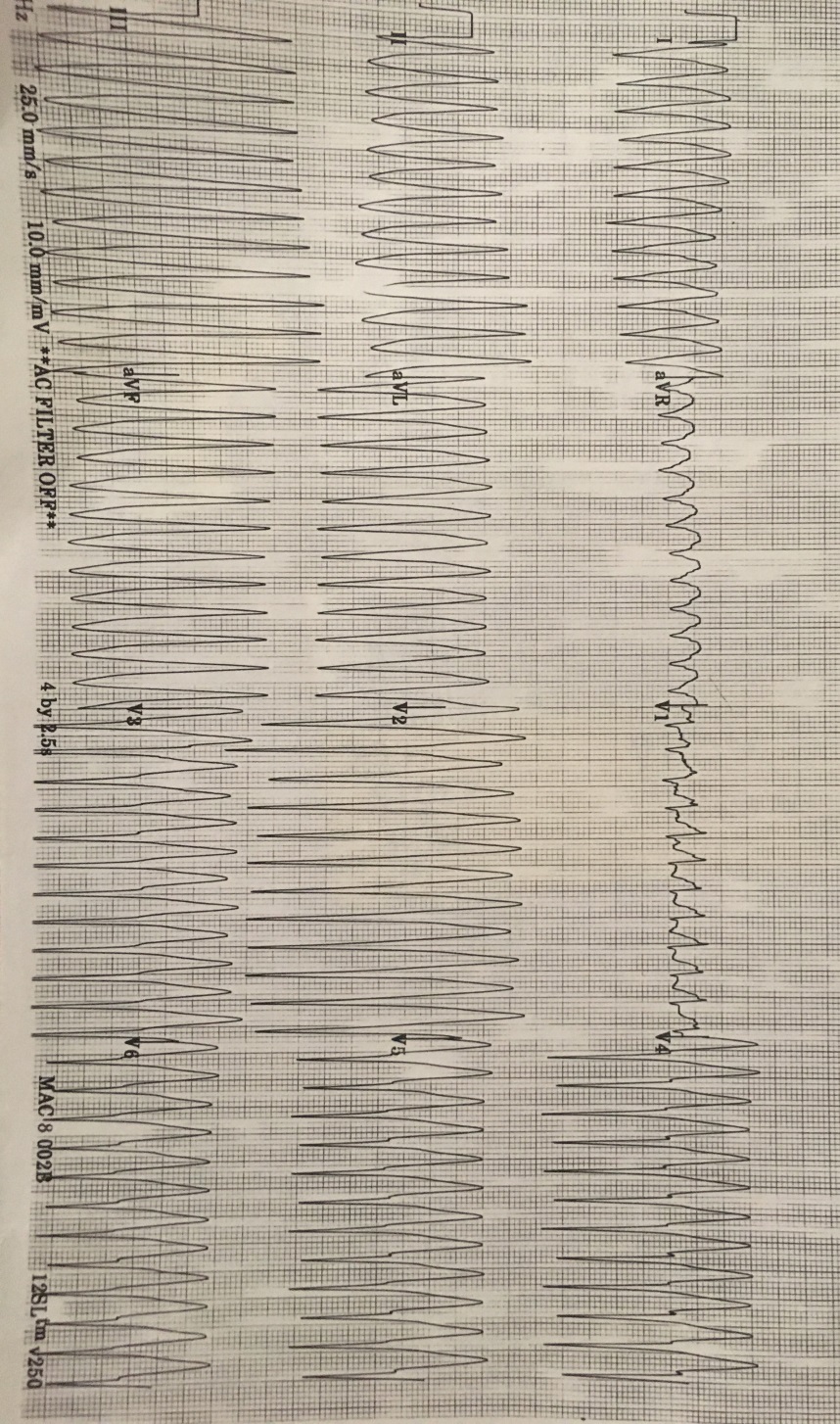


Figure 4: Ventricular Tachycardia EKG. Image courtesy of Dr. Colin Kaide, OSU Emergency Medicine
