# Supplementary material for: Entrustable Professional Activity 10: Case Simulation and Assessment—STEMI With Cardiac Arrest
Source: MedEdPORTAL. 2016 Dec 23;12:10517. doi: 10.15766/mep_2374-8265.10517 (PMC6440413; doi:10.15766/mep_2374-8265.10517)
Supplement: Supplementary file 1 — A. Simulation Case.docx B. Visual Stimuli.docx C. Case Assessment Rubric.docx D. STEMI Management Presentation.pptx [file mep-12-10517-s001.zip › C. Case Assessment Rubric.docx]

**Appendix C:**

**EPA 10: ST Elevation Myocardial Infarction (STEMI) Case Assessment**

| **Student Name** |  |
| --- | --- |
| **Assessor Name** |  |
| **Date:** |  |

| **Critical Actions:** | **Yes** | **No** | **With Prompting** |
| --- | --- | --- | --- |
| Obtains and recognizes patient status and/or unstable vital signs |  |  |  |
| Asks for help when needed from nursing, pharmacy, senior resident, or consultants |  |  |  |
| Determines appropriate disposition for the patient |  |  |  |
| Provides appropriate initial stabilizing treatment for the patient’s acute medical concerns: Obtain 12 lead EKG |  |  |  |
| Provides appropriate initial stabilizing treatment for the patient’s acute medical concerns: Recognize Acute ST Elevation MI |  |  |  |
| Provides appropriate initial stabilizing treatment for the patient’s acute medical concerns: Give Anticoagulation (Antiplatelet Agents and Heparin) |  |  |  |
| Provides appropriate initial stabilizing treatment for the patient’s acute medical concerns: Consult Cardiology for Emergent Catheterization |  |  |  |
| Provides appropriate initial stabilizing treatment for the patient’s acute medical concerns: Performs BLS and ACLS according to protocol for a patient with Pulseless Ventricular Tachycardia |  |  |  |

Would you feel confident in this student’s ability to manage an acutely decompensating/acutely ill patient with a life threatening illness?

- No
- Meets expectations
- Above expectations

**Comments:**
